# The anti-obesity effects of Tongbi-san in a high-fat diet-induced obese mouse model

**DOI:** 10.1186/s12906-018-2420-5

**Published:** 2019-01-03

**Authors:** Yea-Jin Park, Gui-Sun Lee, Se-Yun Cheon, Yun-Yeop Cha, Hyo-Jin An

**Affiliations:** 1 Department of Pharmacology, College of Korean Medicine, Sangji University, 83, Sangjidae-gil, Wonju-si, Gangwon-do 26339 Republic of Korea; 2 Department of Rehabilitative Medicine of Korean Medicine and Neuropsychiatry, College of Korean Medicine, Sangji University, Wonju, Gangwon-do 26339 Republic of Korea; 3 Fubonic Cor. 200 Gieopdosi-ro, Jijeong-myeon, Wonju, Gangwon-do Republic of Korea

**Keywords:** Tongbi-san, High-fat diet, Adipogenesis, Obesity, AMPK

## Abstract

**Background:**

Recently, it has been noted that natural herbal medications may be effective in treating obesity. Tongbi-san (TBS) is a traditional medicine usually used for dysuria (i.e., painful urination), containing three herbs, *Cyperus rotundus* L., *Citrus unshiu* Markovich, and *Poria cocos*. In this study, we aimed to examine whether TBS can inhibit high-fat diet (HFD)-induced adipogenesis in the liver and epididymal adipose tissue of obese mice.

**Methods:**

Male C57BL/6 N mice were fed a normal diet, an HFD, an HFD plus orlistat 10 or 20 mg/kg, or an HFD plus TBS 50 or 100 mg/kg for 11 weeks. Body weight was checked weekly and histological tissue examinations were investigated. An expression of genes involved in adipogenesis was also assessed.

**Results:**

Oral administration of TBS significantly reduced body weight and decreased epididymal and visceral white adipose tissue (WAT) weight. In addition, we found that TBS enhanced the expression of the adenosine monophosphate-activated protein kinase (AMPK) and inhibited the expression of transcription factors, such as CCAAT/enhancer-binding proteins (C/EBPs), sterol regulatory element-binding protein 1 (SREBP1), and peroxisome proliferator-activated receptor γ (PPARγ) in the liver and epididymal WAT as measured by quantitative reverse transcription polymerase chain reaction (qRT-PCR).

**Conclusion:**

These findings demonstrate that the anti-obesity effects of TBS may be linked to the activation of AMPK.

## Background

Obesity is characterized by excessive growth in adipose tissue mass and is rapidly becoming a public health problem that affects millions of people worldwide [1]. Adipose tissue has been considered to be a main regulator of energy homeostasis [2]. Due to an imbalance between energy intake and expenditure, obesity gives rise to excessive growth and expansion of adipose tissue [3]. The white adipose tissue (WAT) is a complex endocrine organ composed of different depots, including subcutaneous (e.g., inguinal) and intra-abdominal (e.g., epididymal and mesenteric) WAT depots [4]. WAT is properly extended to store the surplus energy, however during obesity, it may become severely dysfunctional and fail for these functions. The unhealthy WAT expansion has been correlated with numerous deleterious outcomes, such as inflammation, hypoxia, fibrosis, and disrupted mitochondrial function [5]. Thus, the inhibition of adipose tissue enlargement can be an important target in order to prevent and treat obesity.

The process by which mature adipocytes are generated, adipogenesis, is highly regulated by transcriptional factors including CCAAT/enhancer-binding protein α (C/EBPα), peroxisome proliferator-activated receptor γ (PPARγ), and sterol regulatory element binding protein 1 (SREBP1) [6, 7]. PPARγ is specifically expressed in adipose tissue and acts as a main regulator in adipocyte differentiation and glucose metabolism [8]. C/EBPα is highly expressed in adipose tissue and liver of both rodents and humans [9], and it was reported that C/EBPα-knockout mice miscarry to accumulate lipids in adipocytes [10]. SREBPs adjust the expression of many enzymes involved in synthesis of cholesterol, fatty acids, triacylglycerols, and phospholipids. Consequently, SREBPs regulate cellular lipogenesis and lipid homeostasis [11]. SREBPs are divided into three isoforms: SREBP-1a, SREBP-1c, and SREBP2 [12]. SREBP-1 mainly controls the gene expression involved in fatty acid and triacylglycerol metabolism, while SREBP-2 regulates primarily cholesterol metabolism [13].

The adenosine monophosphate-activated protein kinase (AMPK) is a principal energy sensor, defined as a protein kinase activated by an increase in the AMP/ATP energy ratio [14]. The AMPK is also a heterotrimeric enzyme that plays a master role in energy homeostasis of adipose tissue [15], and is associated with the regulation of C/EBPα and PPARγ [16]. In addition, SREBP1 is also negatively regulated by AMPK [17]. Accordingly, it is likely that the expression of the AMPK is a latent gene target for the suppression of adipogenesis. In this study, we hypothesized that the activation of the AMPK may play a decisive role in a high-fat diet (HFD)-induced mouse model by inhibiting C/EBPα, PPARγ, and SREBP1, thus suppressing adipogenesis.

Recently, it has been noted that natural herbal medications may be an effective treatment for obesity, given that the efficacy and safety of long-term therapy treatment is very important in the management of this life-threatening condition. TBS has been used in traditional Korean medicine to treat conditions including dysuria and circulation dysfunction. In Korean medicine, the theory is, if the body systemic circulation is abnormal, it can manifest itself as an excretion problem or as obesity. Therefore, according to this theory, Korean traditional medical doctors incorporated TBS into the treatment of obesity. TBS consists of *C. rotundus* L., C. *unshiu* Markovich, and *Poria cocos*. The essential oil of *C. rotundus* L. has antioxidant and antibacterial activity against foodborne pathogens [18], and extract of *C. rotundus* L. has been shown to control weight gain in obese Zucker rats [19]. In addition, an herbal extract powder containing C. *unshiu* Markovich has been shown to reduce body fat in overweight adults [20]. Dehydrotrametenolic acid, a compound of *Poria cocos*, was also reported to protect noninsulin-dependent diabetes mellitus in obese mice [21]. For this reason, in the current paper, we investigated the anti-obesity effects of TBS in an HFD-induced obese mouse model.

## Methods

### Chemicals and reagents

TBS consists of *C. rotundus* L., C. *unshiu* Markovich, and *Poria cocos*. The three herbs were purchased from Nanum Pharmaceutical Company (Seoul, Republic of Korea). The herbal samples were performed for sensory test according to ‘The Korean Herbal Pharmacopoeia’ by Prof. Yun-Yeop Cha, and only those that passed the Korean Pharmacopoeia standard were selected and used for this experiment. TBS was made using a 1: 1: 1 ratio of these herbs (400 g each). The herbs were then extracted in water at 99 °C for 3 h. The extract was freeze-dried, and the yield rate was calculated at 33.20% (33.20 g per 100 g of liquid extract). The powder was dissolved in distilled water for this experiment, and the residual powder was stored at − 20 °C. The 30% HFD was obtained from Research Diets (New Brunswick, NJ, USA). The p-AMPK and AMPK antibodies were obtained from Cell Signaling Technology (Danvers, MA, USA). PPARγ, C/EBPα, SREBP1, AMPK, and glyceraldehyde-3-phosphate dehydrogenase (GAPDH) oligonucleotide primers were purchased from Bioneer Corporation (Daejeon, Republic of Korea), and SYBR Premix Ex Taq was purchased from Takara Bio Inc. (Otsu, Japan). Orlistat was purchased Tokyo Chemical Industry Co. Ltd. (Tokyo, Japan) and other reagents were purchased from Sigma-Aldrich Co. LLC (St. Louis, MO, USA).

### HFD-induced obesity mouse model

Eight-week-old male C57BL/6 N mice (specific-pathogen-free (SPF) grade, 20 ± 2 g) were purchased from Daehan Biolink (Daejeon, Republic of Korea). Prior to the start of the experiment, mice were adapted to the modified conditions for 1 week and 36 healthy mice were used in this study. Mice were then randomly distributed into six groups (*n* = 6 per cage): the normal diet group (CON), 30% high fat-diet group (HFD), orlistat-administered groups (orlistat 10 or 20 mg/kg orally [p.o.]), and TBS-administered groups (TBS 50 or 100 mg/kg p.o.). The mice were given free approach to food and water. With the exception of the CON group, all of the other mice were fed an HFD. TBS- or orlistat-treated groups were administered TBS or orlistat orally, whereas the other groups were treated with physiological saline. Body weight and food intake were recorded every week and they were maintained under a 12 h light/dark cycle at a constant temperature of 22 ± 2 °C with a relative humidity of 55 ± 9%. At the end of an 11-week period, all animals were fasted for 12 h, anaesthetized with Zoletil 50 (20 mg/kg) administered intraperitoneally according to the manufacturer’s instruction (Virbac, Carros Cedex, France), and euthanized by cervical dislocation. The liver and adipose tissues were then taken, rinsed, weighed, and directly stored at − 80 °C until further analysis. All procedures were conducted in accordance with the National Institute of Health guidelines and approved by the Ethical Committee for Animal Care and the Use of Laboratory Animal of Sangji University (reg.no. 2017–12).

### Serum analysis

At the end of each experiment, the blood samples of treated mice were collected and centrifuged at 1000×*g* for 20 min. The collected serum concentration was used to determine total cholesterol (TC), blood urea nitrogen (BUN), aspartate aminotransferase (AST), and alanine aminotransferase (ALT) using enzymatic methods from commercially available kits (BioVision; Milpitas, CA, USA).

### Histological analysis

The liver and epididymal adipose tissue from a representative mouse in each group were fixed in 10% buffered formalin, embedded in paraffin, and cut into 8 μm thick sections. Some sections were stained with hematoxylin and eosin (H&E) for histological examination of lipid droplets and images were acquired using an Olympus SZX10 microscope (Tokyo, Japan).

### Western blot analysis

Segments of liver or epididymal adipose tissue were suspended in PRO-PREP™ protein extraction solution (Intron Biotechnology, Seoul, Republic of Korea) and incubated for 20 min at 4 °C. Cell debris was removed via micro-centrifugation, followed by quick freezing of the supernatant. The protein concentration was determined using the Bio-Rad protein assay reagent (Bio-Rad, Hercules, CA, USA) according to the manufacturer’s instructions. Cellular proteins from treated and untreated cell extracts were electroblotted onto a polyvinylidene fluoride membrane following separation via 10–12% SDS-PAGE. The blot was incubated for 1 h with blocking solution (5% skim milk) at room temperature, followed by overnight incubation with primary antibody (1:1000) at 4 °C. Blots were washed three times with Tween 20/Tris-buffered saline (T/TBS) and incubated with horseradish peroxidase-conjugated secondary antibody (1:2000) for 2 h at room temperature. Blots were again washed three times with T/TBS, and then developed via enhanced chemiluminescence (GE Healthcare, Waukesha, WI, USA). Densitometric analysis was performed using Bio-Rad Quantity One Software.

### Quantitative real-time polymerase chain reaction (qRT-PCR) analysis

The liver and epididymal WAT were homogenized, and the total RNA was isolated using the Easy-Blue® reagent according to the manufacturer’s instructions (Intron Biotechnology; Seongnam, Republic of Korea). The cDNA was synthesized according to a previously reported procedure [22]. The oligonucleotide primers for mouse PPARγ were ATCGAGTGCCGAGTCTGTGG (forward) and GCAAGGCACTTCTGAAACCG (reverse); for mouse C/EBPα were GGAACTTGAAGCACAATCGATC (forward) and TGGTTTAGCATAGACGTGCACA (reverse); for mouse SREBP1 were ATCGCAAACAAGCTGACCTG (forward) and AGATCCAGGTTTGAGGTGGG (reverse); for mouse AMPK were GGTGGATTCCCAAAAGTGCT (forward) and AAGCAGTGCTGGGTCACAAG (reverse); for mouse GAPDH were GACGGCCGCATCTTCTTGT (forward) and CACACCGACCTTCACCATTTT (reverse). Gene expression was calculated according to the comparative threshold cycle (Ct) method.

### Statistical analysis

Each result is expressed as the mean ± standard deviation (SD) of triplicate experiments. Statistical analysis was fulfilled using SPSS statistical analysis software (version 19.0; International Business Machines, Armonk, NY, USA). Statistically significant differences were determined using analysis of variance and Dunnett’s post hoc test, and *P*-values of less than 0.05 were considered statistically significant.

## Results

### TBS suppressed adipose tissue size and body weight in mice with HFD-induced obesity

**Fig. 1:**
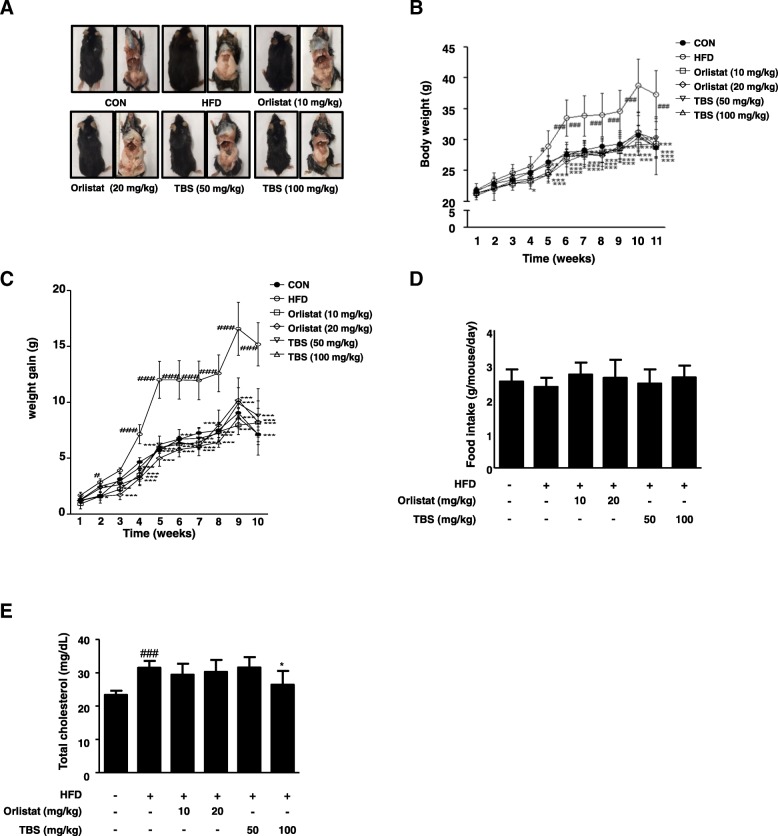
Effect of TBS on adipose tissue size and body weight in high-fat diet-induced obesity in C57BL/6 N mice. (**a**) Macroscopic mouse body and WAT size analysis. (**b**) Body weight and (**c**) body weight gain were assessed every week. (**d**) Food intake was recorded two or three times per one week. (**e**) The levels of serum TC were determined using enzymatic methods. CON: normal diet group; HFD: high-fat diet group; Orlistat: HFD plus orlistat (10 or 20 mg/kg) group; TBS: HFD plus TBS (50 or 100 mg/kg) group. The values represent the mean ± S.D. ^#^*p* < 0.05 and ^###^*p* < 0.001 vs. the control group; ^*^p < 0.05, ^**^p < 0.01, and ^***^p < 0.001 vs. HFD group

After 1 week of adaption, the animals used for this study were randomly distributed into six groups (*n* = 6): the normal diet (CON) group, the high-fat diet (HFD) group, the HFD plus orlistat 10 mg/kg group (Orlistat 10 mg/kg), the HFD plus orlistat 20 mg/kg group (Orlistat 20 mg/kg), the HFD plus TBS 50 mg/kg group (TBS 50 mg/kg), and the HFD plus TBS 100 mg/kg group (TBS 100 mg/kg). Compared with the CON mice, the mice fed an HFD presented significantly increased fat mass to the naked eye after the eleventh week. Oral administration of TBS at 50 or 100 mg/kg decreased the fat mass compared with that of the HFD group (Fig. 1a). Body weight was measured every week and there was a significant difference between the CON group and the HFD group. Notably, the TBS-treated groups had lower weight compared with the HFD group (Fig. 1b). Body weight gain in the HFD group also remarkably increased compared with that of the CON group. The HFD group gained a total average of 15.18 ± 1.93 g, whereas the TBS treated groups (50 or 100 mg/kg) gained only 8.81 ± 1.32 or 7.16 ± 0.95 g, respectively (Fig. 1c). However, there was no significant difference of food intake in the HFD group compared with that of the other groups during the experimental period (Fig. 1d). In addition, there were no signs of pathology or abnormalities in mice administered TBS. The concentrations of serum TC in the HFD group were significantly increased compared with that of the CON group. The level of serum TC was 31.50 ± 2.07 mg/dL in the mice fed an HFD, whereas the treatment of TBS at 100 mg/kg reduced the TC level to 26.44 ± 4.13 mg/dL. This results showed a better result in TC level reduction than orlistat-treated groups (Fig. 1e). To determine the effect of TBS on liver and kidney tissue, we assessed the amount of BUN, ALT, and AST in the blood serum. The levels of BUN represent kidney toxicity, whereas the levels of ALT and AST stand for liver toxicity. Serum samples were prepared from each group and BUN, AST, and ALT levels were analyzed by enzymatic methods. The levels of BUN, AST, and ALT were within normal range and there were no significant increases of these enzymes in TBS-treated groups according to the blood serum analysis (Table 1).

**Table 1:** Effect of TBS administration on blood biochemistry in HFD-induced mice

	Groups		
Parameters	BUN (mg/dL)	AST (U/L)	ALT (U/L)
CON	4.29 ± 0.61	37.00 ± 5.93	7.90 ± 1.97
HFD	3.98 ± 0.40	34.25 ± 9.47	7.38 ± 1.77
Orlistat (10 mg/kg)	3.83 ± 0.37	30.14 ± 1.46	5.22 ± 0.67
Orlistat (20 mg/kg)	3.87 ± 0.38	38.33 ± 5.51	5.40 ± 0.55
TBS (50 mg/kg)	3.78 ± 0.32	36.00 ± 6.98	5.50 ± 0.85
TBS (100 mg/kg)	4.20 ± 0.63	37.5 ± 7.26	5.33 ± 0 .52

The values are represented as mean ± S.D. (*n* = 6). Abbreviations are: *BUN* blood urea nitrogen, *AST* aspartate aminotransferase, *AST* alanine aminotransferase

### TBS suppressed total fat mass in mice with HFD-induced obesity

**Fig. 2:**
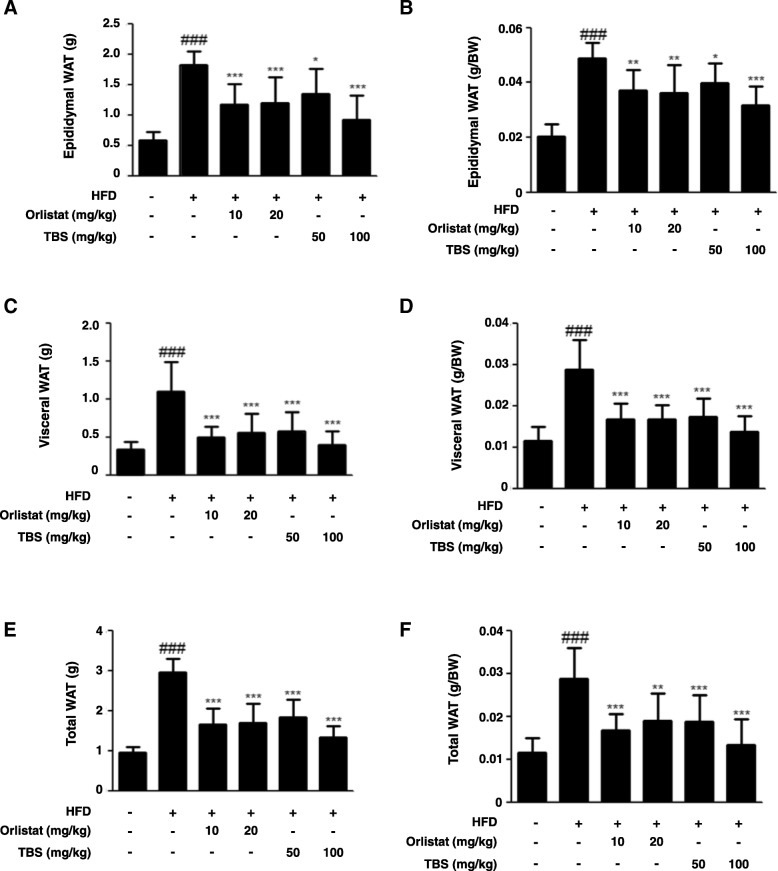
Effect of TBS on adipose tissue weight in high-fat diet-induced obesity in C57BL/6 N mice. (**a**) The epididymal adipose tissue weight, (**b**) relative epididymal adipose tissue weight ratio, (**c**) visceral adipose tissue weight, (**d**) relative visceral adipose tissue weight ratio, (**e**) total adipose tissue weight, and (**f**) total adipose tissue weight ratio were measured after 11 weeks of diet treatments in mice. The values represent the mean ± S.D. ^###^*p* < 0.001 vs. the control group; ^*^p < 0.05, ^**^p < 0.01, and ^***^p < 0.001 vs. HFD group

The WAT mass is influenced by adipogenesis, the underlying process of pre-adipocyte differentiation into mature adipocytes [23]. As shown in Fig. 2a and b, there was a significant difference in epididymal WAT weight between the CON group and the HFD group. The TBS-treated groups had significantly lower weights than the HFD group. Moreover, there was also a remarkable difference in visceral WAT weight between the CON group and the HFD group. TBS-treated groups showed significantly lower weight than the HFD group (Fig. 2c and d). The mass of epididymal, visceral, and total WAT declined by 59.56, 64.17, and 55.03%, respectively, in the TBS (100 mg/kg) treated group compared with those of the HFD group (Fig. 2).

### TBS suppressed HFD-induced lipid accumulation in the epididymal adipose tissue

**Fig. 3:**
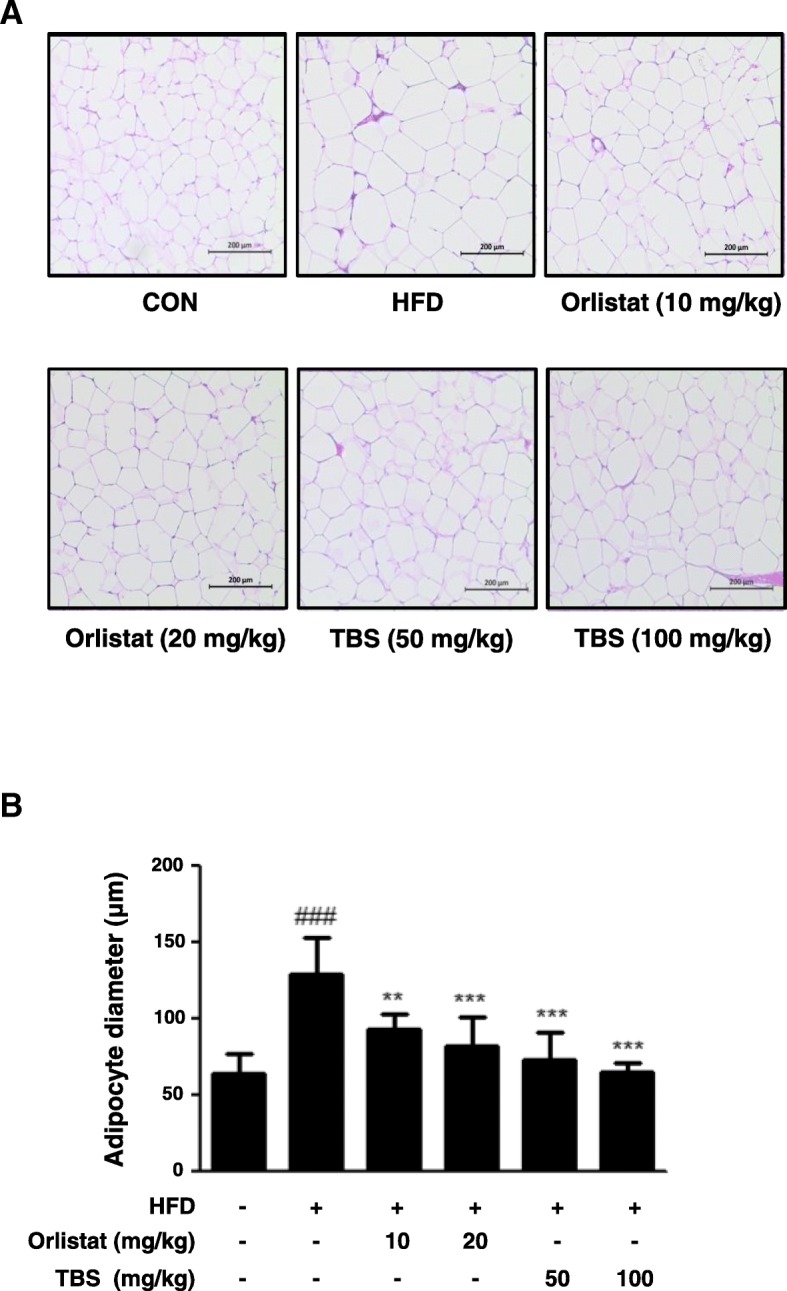
Effects of TBS on lipid accumulation in epididymal white adipose tissue. **(a**) The epididymal white adipose tissue (WAT) from representative mice in each group was fixed, embedded in paraffin, and stained with H&E. Images are shown at the original magnification of 100x. (**b**) The average diameter of adipocytes in epididymal WAT of each group. CON: normal diet group; HFD: high-fat diet group; Orlistat: HFD plus orlistat (10 or 20 mg/kg) group; TBS: HFD plus TBS (50 or 100 mg/kg) group. The values represent the mean ± S.D. of three independent experiments. ^###^*p* < 0.001 vs. the control group; ^**^p < 0.01 and ^***^p < 0.001 vs. HFD group. Scale bar is 200 μm

To identify the effects of TBS on HFD-induced lipid accumulation in epididymal WAT, adipose tissue samples were prepared from each group and stained with H&E solution. As shown in Fig. 3a, the H&E staining results for epididymal WAT from each group, and data revealed that lipid accumulation in epididymal WAT increased remarkably in the HFD group compared with the CON group. However, TBS administration significantly decreased lipid accumulation in the HFD-fed mice. Secondly, the average diameter of adipocytes in epididymal WAT increased in HFD-fed mice and TBS treatment significantly lowered the adipocyte diameter of these mice (Fig. 3b).

### TBS suppressed HFD-induced expression of adipogenesis-related genes in the epididymal adipose tissue

**Fig. 4:**
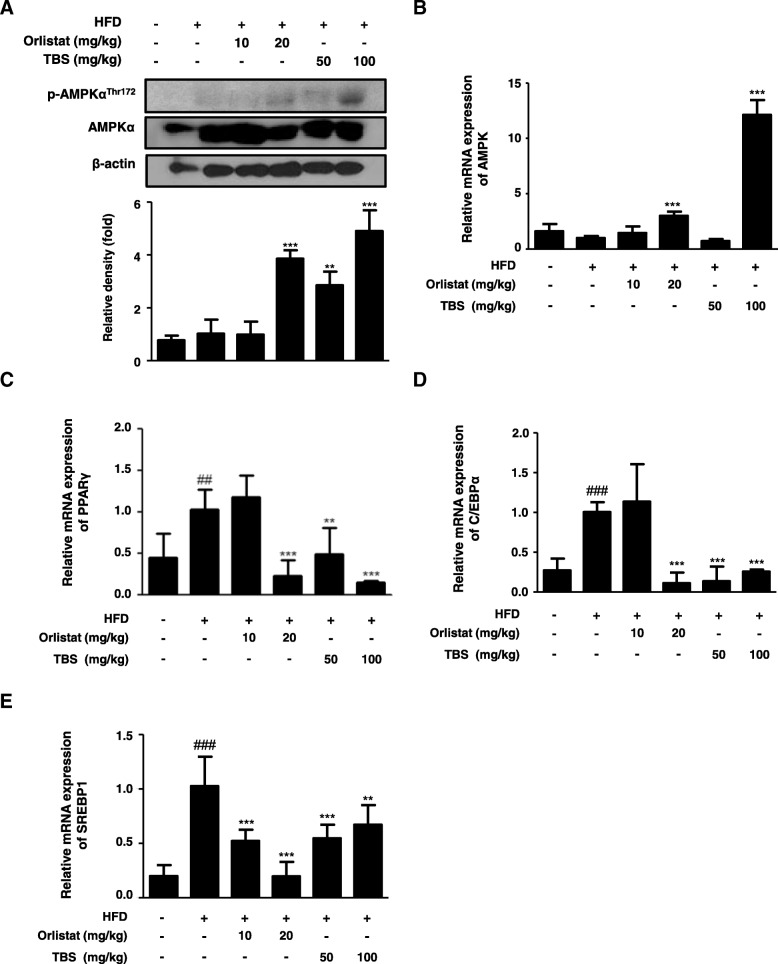
Effects of TBS on AMPK, PPARγ, C/EBPα, and SREBP1 mRNA expression in epididymal white adipose tissue. Total protein and RNA were prepared from epididymal white adipose tissue (WAT), and the protein expression of (**a**) p-AMPK and the mRNA levels of (**b**) AMPK, (**c**) PPARγ, (**d**) C/EBPα, and (E) SREBP1 were determined by western blot analysis and quantitative RT-PCR (qRT-PCR). Densitometric analysis was performed using ImageJ ver. 1.50i. The values represent the mean ± S.D. of three independent experiments. ^##^*p* < 0.01 and ^###^ *p* < 0.001 vs. the control group; ^**^p < 0.01 and ^***^p < 0.001 vs. HFD group

To investigate the effects of TBS on the protein expression of the p-AMPK and mRNA levels of the AMPK, PPARγ, C/EBPα, and SREBP1 in the epididymal WAT, western blotting and qRT-PCR were also performed. As shown in Fig. 4a, there was no difference of the protein expression of the AMPK Thr172 phosphorylation in the HFD group compared with that of the CON group, whereas TBS-treated groups significantly enhanced the expression of p-AMPK^Thr172^ in epididymal WAT. Likewise, TBS treatment at a dose of 100 mg/kg significantly increased the mRNA level of AMPK in epididymal WAT (Fig. 4b). Furthermore, there was an increase in the mRNA expression of PPARγ, C/EBPα, and SREBP1 of the HFD group compared with the CON group, whereas administration of orlistat and TBS effectively decreased the mRNA expression of PPARγ, C/EBPα, and SREBP1 in epididymal WAT (Fig. 4c, d, and e).

### TBS suppressed HFD-induced lipid droplet accumulation in liver tissue

**Fig. 5:**
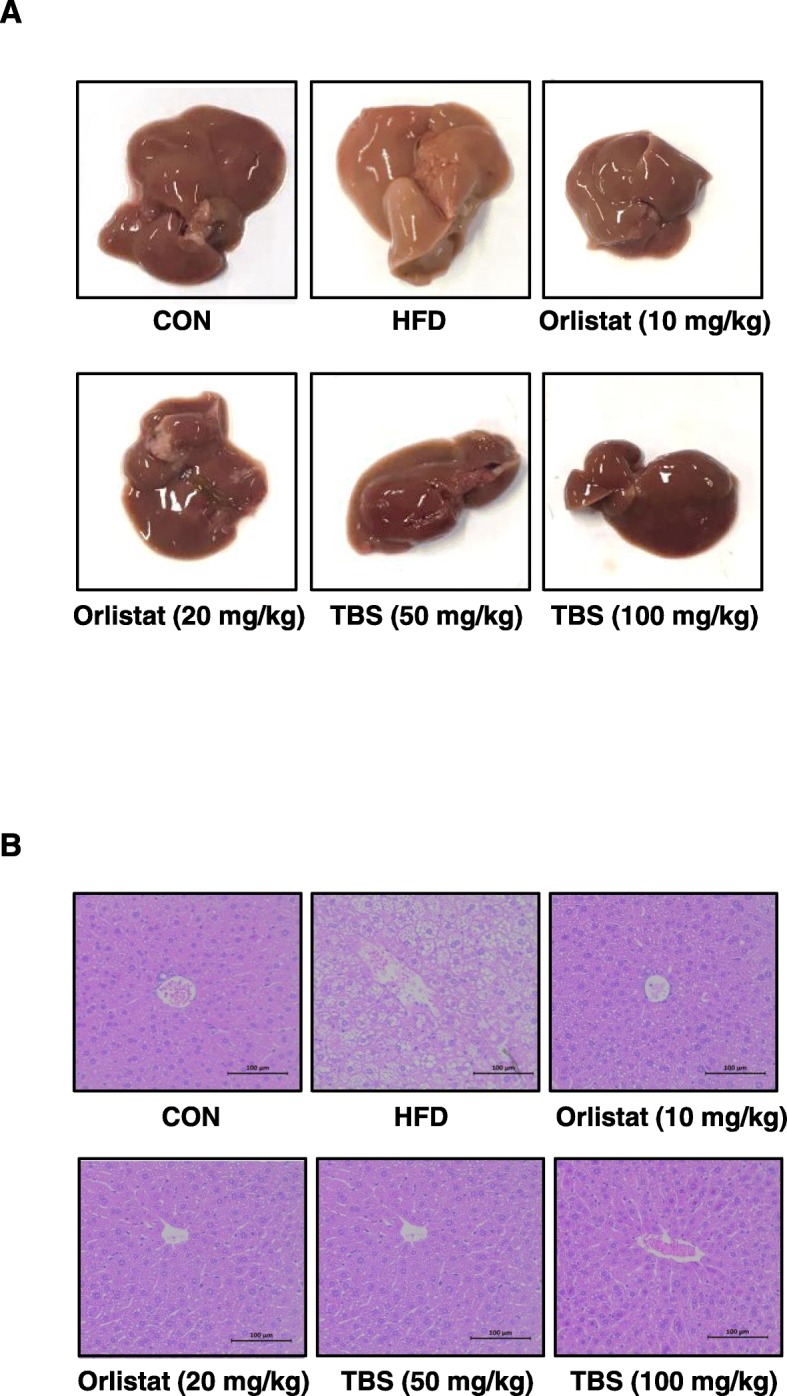
Effects of TBS on liver tissue morphology and lipid accumulation. (**a**) Macroscopic analysis of mouse liver tissue. (**b**) Liver tissue from representative mice in each group were fixed, embedded in paraffin, and stained with H&E solution. Images are shown at the original magnification of 100x. CON: normal diet group; HFD: high-fat diet group; Orlistat: HFD plus orlistat (10 or 20 mg/kg) group; TBS: HFD plus TBS (50 or 100 mg/kg) group. Scale bar is 100 μm

Li et al. have reported that HFD feeding markedly lightened the color of the liver in a non-alcoholic fatty liver disease rat model [24]. To determine the effect of TBS in the liver tissue morphology, tissue samples were observed using macroscopic analysis. As shown in Fig. 5a, the liver tissue of mice in the HFD group turned pale compared with that of mice in the CON group. Oral administration of TBS (50 or 100 mg/kg) significantly suppressed the morphological change observed in the liver tissue compared with that of the HFD group. Orlistat-treated groups also presented recovered liver tissue morphology. In addition, tissue samples were stained with H&E. As shown in Fig. 5b, in the HFD group, lipid droplets appeared as small vacuoles within liver cells. Enlargement of lipid droplets were more pronounced in the liver tissue of mice belonging to the HFD group than that of the TBS-treated groups (50 or 100 mg/kg).

### TBS suppressed HFD-induced expression of adipogenesis-related genes in liver tissue

**Fig. 6:**
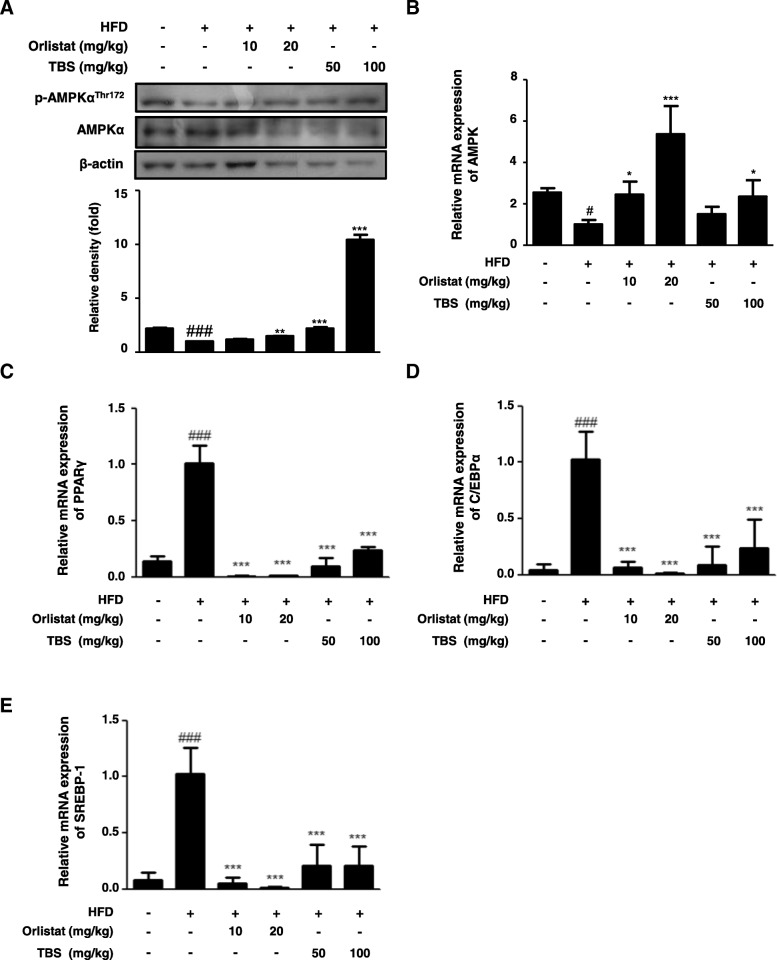
Effects of TBS on liver tissue the expression of p-AMPK, AMPK, PPARγ, C/EBPα, and SREBP1. Total protein and RNA prepared from liver tissue were used to measure the expression of protein of (**a**) p-AMPK and the levels of mRNA of (**b**) AMPK, (**c**) PPARγ, (**d**) C/EBPα, and (**e**) SREBP1 by western blotting and qRT-PCR. Densitometric analysis was performed using ImageJ ver. 1.50i. The values represent the mean ± S.D. of three independent experiments. ^#^*p* < 0.05 and ^###^*p* < 0.001 vs. the control group; ^*^p < 0.05, ^**^p < 0.01, and ^***^p < 0.001 vs. HFD group

The AMPK acts a master switch, which phosphorylates target enzymes associated with lipid metabolism in many tissues, including the liver [25]. The expression of PPARγ, C/EBPα, and SREBP1 is induced in the liver of HFD-induced mice [26–28]. In this study, the protein expression of hepatic p-AMPK and mRNA levels of hepatic AMPK, PPARγ, C/EBPα, and SREBP1 were examined in the liver of mice fed an HFD plus TBS by western blotting and qRT-PCR, respectively. The protein expression of the AMPK Thr172 phosphorylation in the liver of the HFD group was diminished when compared with the CON group and TBS-treated groups enhanced the expression of the p-AMPK^Thr172^ (Fig. 6a). Similarly, treatment of TBS (100 mg/kg) significantly increased the mRNA level of AMPK in liver tissue (Fig. 6b). Moreover, the mRNA expression of PPARγ, C/EBPα, and SREBP1 was up-regulated in the HFD group compared with that in the CON group, and administration of orlistat and TBS considerably inhibited the mRNA expression of hepatic PPARγ, C/EBPα, and SREBP1 (Fig. 6c, d, and e).

## Discussion

Obesity is a multifactorial disease characterized by a superfluity of adiposity and an overconsumption of dietary fat [29]. It is also characterized as a state of chronic inflammation and adipose tissue hypoxia resulting in dysregulation in adipokine production and activation of pro-inflammatory pathways, which can promote tumor progression [30]. Obesity-related diseases have become the main cause of death in modern societies and over-nutrition has been found to be associated with complex types of cancers [31]. Accordingly, it is necessary to treat obesity as a source of many diseases. The common side effects of conventional pharmacological treatments for obesity and related conditions, such as hypertension, cardiac arrhythmia, constipation, headache, steatorrhea, and deficiencies of lipid soluble vitamins and essential fatty acids, have contributed to the increased use of traditional herbal medicine as a healthcare modality for this life-threatening condition [32]. Consequently, Natural compounds that are relatively safe are receiving attention in treating obesity. In the present study, we aimed to assess the effects of TBS on body weight and the expression of related adipogenesis genes in high fat-diet induced obese mice.

TBS is one of the traditional Asian medicine prescriptions excerpted from a representative Korean medical book, Donguibogam, and it has been widely used to treat dysuria, such as the infection of urinary system, calculus, chyluria, and hematuria. *Cyperus rotundus* L. and *Citrus unshiu* Markovich, the major components of TBS, have also been reported to be beneficial in preventing weight gain and obesity, respectively [19, 20]. Accordingly, the active components of these three herbs may be associated with the powerful effect of TBS in HFD-fed obese mice.

It has been reported that an HFD is a risk factor resulting in an increase of whole-body fat and fat distribution, particularly the accumulation of visceral adipose tissue [33], as well as serum level of TC [34, 35]. Hence, an HFD-induced obese mouse model was used to evaluate the anti-obesity properties of TBS. Body weight and fat weight increase is a warning sign of overall health status [36]. Our study demonstrated that TBS-treated groups had significantly lower body weight than that in the HFD only group (Fig. 1b and c). However, the food intake of TBS treated groups was not any different from the other groups (Fig. 1d). TBS treatment at 100 mg/kg also abolished the increased TC level in serum of the mice fed HFD (Fig. 1e). In addition, we examined blood serum for BUN, AST, and ALT levels. AST and ALT levels are used to assess liver damage, while serum levels of BUN are used to determine kidney damage. Our blood serum analysis demonstrated that TBS-treated groups did not show a significant increase in the levels of these enzymes (Table 1). It has been reported that epididymal WAT can regulate whole body homeostasis as well as enhance immunity and inflammation by secreting various adipokines [37]. Previous studies reported that visceral WAT is a major inflammatory adipose tissue in HFD-induced obesity [38]. Our results revealed that TBS strongly decreased epididymal and visceral WAT in comparison with HFD-induced obese mice and showed a better result in epididymal and visceral fat mass reduction than orlistat-treated groups (Fig. 2). Orlistat was used as a positive control because of its effectiveness in managing weight by reducing leptin levels and fat mass [39]. Orlistat changes the amount of fat delivered to the liver as well as the type of fat, thereby modulating insulin action to reduce the absorption of dietary fat [40]. In addition, TBS administration inhibited epididymal adipocyte size in HFD-induced adipose tissue gain in a dose-dependent manner (Fig. 3). These results indicated that TBS is capable of reducing lipid accumulation in epididymal WAT better than orlistat.

The liver is mostly regarded as an essential organ in lipid metabolism. Imbalance between lipid deposition and removal results in hepatic lipid accumulation, which is related to increased hepatic lipogenesis, augmented lipid uptake and/or declined triglyceride export of β-oxidation [41]. The liver tissue of mice in the HFD group turned pale, while TBS-treated groups improved this sign of liver tissue toxicity (Fig. 5a). As shown in Fig. 5b, lipid accumulation was highly induced in mice fed an HFD, but it was inhibited in mice treated with TBS as seen in liver tissue lipid droplet reduction. This means that the liver of TBS-treated mice had less adipocytes than the HFD-fed mice. These findings highlight the fact that TBS is also competent in reducing lipid accumulation in liver tissue, as well as epididymal WAT.

The AMPK is a heterotrimeric complex including one catalytic α-subunit and two regulatory β- and γ-subunits [42]. Activation of AMPK occurs when AMP binds to the nucleotide binding site on the γ-subunit, leading to a conformational change in the α-subunit allowing the upstream kinases, such as LKB1, to phosphorylate AMPK at threonine-172 [43]. Thus, AMPK is a phylogenetically conserved serine/threonine kinase that mediates cellular energy homeostasis via the enzymatic activity triggered by phosphorylation of threonine-172 [44]. In addition, the AMPK has been implicated in the regulation of glucose and lipid homeostasis in hepatocytes [45], and once AMPK is activated, lipogenesis in liver is inhibited, which subsequently suppresses fat accumulation [16]. Moreover, AMPK activation in adipose tissue inhibits PPARγ and adipogenesis, thereby reducing fat accumulation [46]. Because obesity is a disorder related to energy imbalance, the AMPK, a crucial cellular energy sensor [47], can be a major target for treating obesity. With the above points in mind, in this study, we examined protein expression of the AMPK Thr172 phosphorylation and mRNA level of AMPK in the epididymal adipose tissue and liver (Figs. 4a, b, 6a, and b). TBS treatment was up-regulated the protein expression of p-AMPK^Thr172^ and mRNA level of AMPK in the epididymal adipose tissue and liver. Both TBS and orlistat are effective in suppressing lipid accumulation in the epididymal adipose tissue and liver. More precisely, TBS treatment is more effective in the epididymal adipose tissue, because orlistat primarily aims to prevent absorption of fat transferred to the liver and TBS may be directly preventing the absorption of fat into epididymal adipose tissue. Several reports have demonstrated that PPARγ, C/EBPα, and SREBP1 are important transcriptional genes involved in adipogenesis [47]. The HFD stimulates expression of PPARγ and C/EBPα, which work in a self-adjusting positive feedback loop system to increase the expression of genes related to adipogenesis and activate the expression of lipid-metabolizing enzymes, resulting in morphological changes and lipid accumulation in cells [48]. The mature forms of SREBPs are transcriptionally activated and are translocated to the nucleus where they bind to the promoters of SREBP target genes, the majority of which are touched on lipid metabolism [49]. Our findings demonstrated that TBS could repress adipogenesis by regulating AMPK, PPARγ, C/EBPα, and SREBP1 expressions in an HFD-fed obese mouse model (Figs. 4 and 6). Therefore, it may also be possible that TBS exerts anti-obesity effects through the regulation of these transcription factors.

**Fig. 7:**
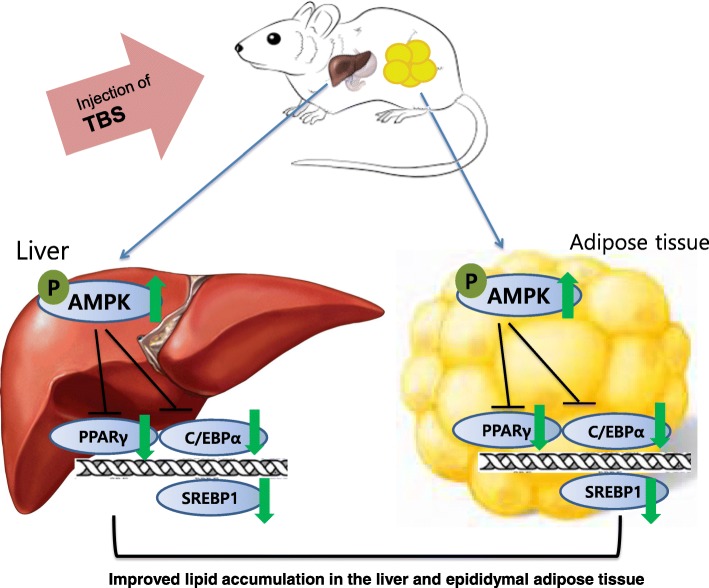
The mechanism of TBS. TBS injections significantly reduced lipid accumulation by regulating the expression of AMPK, PPARγ, C/EBPα, and SREBP1 in the liver and epididymal white adipose tissue of an HFD-induced obese mouse model

Taken together, our findings showed that TBS effectively inhibited lipid accumulation in the liver and epididymal adipose tissue. The recovery of AMPK, PPARγ, C/EBPα, and SREBP1 expression is involved in the mechanism underlying the anti-adipogenesis effects of TBS (Fig. 7). Taken into consideration all of these, further studies may contribute to our current grasp of TBS efficacy and the use of TBS as a beneficial candidate for the control of obesity should be explored further.

## Conclusions

Our findings suggest that TBS treatment could repress adipogenesis by regulating lipid accumulation and adipogenic-related factors in HFD-induced obese mice. Thus, TBS has an inhibitory activity on adipogenesis and a role as potential therapeutic agent for obesity.

## Abbreviations

ALT: Alanine aminotransferase
AMPK: Adenosine monophosphate-activated protein kinase
AST: Aspartate aminotransferase
BUN: Blood urea nitrogen
C/EBPα: CCAAT/enhancer-binding protein α
HFD: High-fat diet
PPARγ: Peroxisome proliferator-activated receptor γ
SREBP1: Sterol regulatory element-binding protein 1
TBS: Tongbi-san
WAT: White adipose tissue
